# Beyond the black swan: climate change and the evolving risk of multi-mycotoxin contamination

**DOI:** 10.3389/ffunb.2026.1981215

**Published:** 2026-10-14

**Authors:** Angel Medina

**Affiliations:** Department of Food Biotechnology, Institute of Agrochemistry and Food Technology (IATA-CSIC), Paterna, Valencia, Spain

**Keywords:** climate change, CO2, ecological niche, food safety, fungal ecology, multi-mycotoxin contamination, mycotoxins

## Abstract

Major mycotoxin contamination events have traditionally been treated as “black swans,” rare, high-impact episodes that are unpredictable in advance and explained only after they occur. This framing assumes long intervals between events and a single dominant toxin per episode, an assumption that reflects, in part, historical single-analyte monitoring rather than proof that other toxins were biologically absent. Climate change is a plausible driver of change in both assumptions. As interacting climate factors, temperature, water availability and atmospheric CO_2_, become more frequent and more extreme, episodes that used to be spaced by decades could increasingly occur several times within the same cropping season. This would not only be a change in frequency. The same interacting factors have been shown, in experimental systems, to modify, and in many cases widen, the range of conditions under which mycotoxigenic fungi grow and, at the same time, the range of conditions under which they produce toxin, so that windows that used to be separate can overlap for several species at once. The consequence, consistent with survey data across several crops, food products and continents, though improved multi-analyte detection likely also contributes to it, may not be simply more of one toxin but the regular co-occurrence of multiple mycotoxins in the same raw material. Current risk models, surveillance programs and legislation remain largely built around single-toxin, single-species assumptions calibrated on historical, regional data. This Perspective proposes an ecological framework linking climate change to an increased risk of multi-mycotoxin contamination, offered as a plausible hypothesis rather than an established transition, and outlines what this implies for monitoring, modeling and regulation.

## 1 The mycotoxin outbreak as a black swan

For decades, major mycotoxin contamination episodes have been described as black swans, rare and unpredictable events explained only in hindsight, even where the underlying drivers were not, strictly, unforeseeable (Taleb, 2007). A single hot, dry season pushing aflatoxin B1 in maize far above baseline; an unusually wet harvest triggering a deoxynivalenol outbreak in wheat. These episodes were treated as exceptions, separated by years or decades and dominated by a single toxin in a single crop.

This framing carried three implicit assumptions. That such events are rare, with long return intervals; that each is explainable by one dominant environmental trigger, a drought year, a wet harvest; and that each is associated with one dominant toxin and, usually, one dominant fungal species. Part of this picture reflects genuine biology, but part of it also reflects the analytical and regulatory conventions of the time, when routine monitoring targeted one or a few regulated mycotoxins rather than the broad multi-analyte panels available today, so co-occurring toxins were often not sought and therefore not reported; we return to this detection-versus-occurrence distinction in Section 4. With that caveat, these assumptions were reasonable when climate anomalies were infrequent and are becoming less so as climate change alters the frequency and ecological consequences of the conditions that drive mycotoxin contamination.

This Perspective argues that two of these assumptions, rarity and single-toxin dominance, are breaking down together and are mechanistically linked. The same interacting climate factors that increase the frequency of extreme conditions also widen the ecological space available to mycotoxigenic fungi, in time within a single season and in space across latitudes. The result is not a string of isolated black swans, but their compounding into multi-toxin contamination as the normal state of a raw material. Because the same crops, climatic drivers and raw materials also feed livestock and cycle through the wider environment, this shift carries important implications for One Health, extending the risk beyond food safety to animal and environmental health.

## 2 From isolated events to overlapping events within a single season

Work on climate change and mycotoxins has, since the early 2000s, focused on the combined effects of temperature, water availability and elevated CO_2_, rather than any one factor alone (Magan et al., 2011; Medina et al., 2014, 2015), because single-factor studies consistently mis-estimated fungal growth and mycotoxin production relative to combined-factor data (Medina et al., 2017).

What has changed is the timescale over which these factors vary. Extreme temperature, rainfall and drought episodes are becoming more frequent within the same region (IPCC, 2022; Casu et al., 2024), sometimes within a single cropping season rather than spread across successive years, so a season that once brought a single anomalous event can now bring several in sequence, an early heat spike, a mid-season drought, a late intense rainfall event. Pests and pathogens more broadly are already shifting polewards at a mean rate of 2.7 km per year since 1960 (Bebber et al., 2013; Bebber, 2015), a shift paralleled by the climatically suitable ranges of crops themselves (Pritchard, 2011) and by temperature-dependent infection risk for fungal crop pathogens, projected to rise fastest at higher, increasingly productive latitudes (Chaloner et al., 2021).

A framework built around single, spaced-out anomalies cannot describe a regime in which several occur in succession within one season, in the same field (**Figure 1A**). This is the central shift this Perspective addresses, not simply more frequent extremes but extremes that now cluster within a single crop cycle.

**Figure 1:**
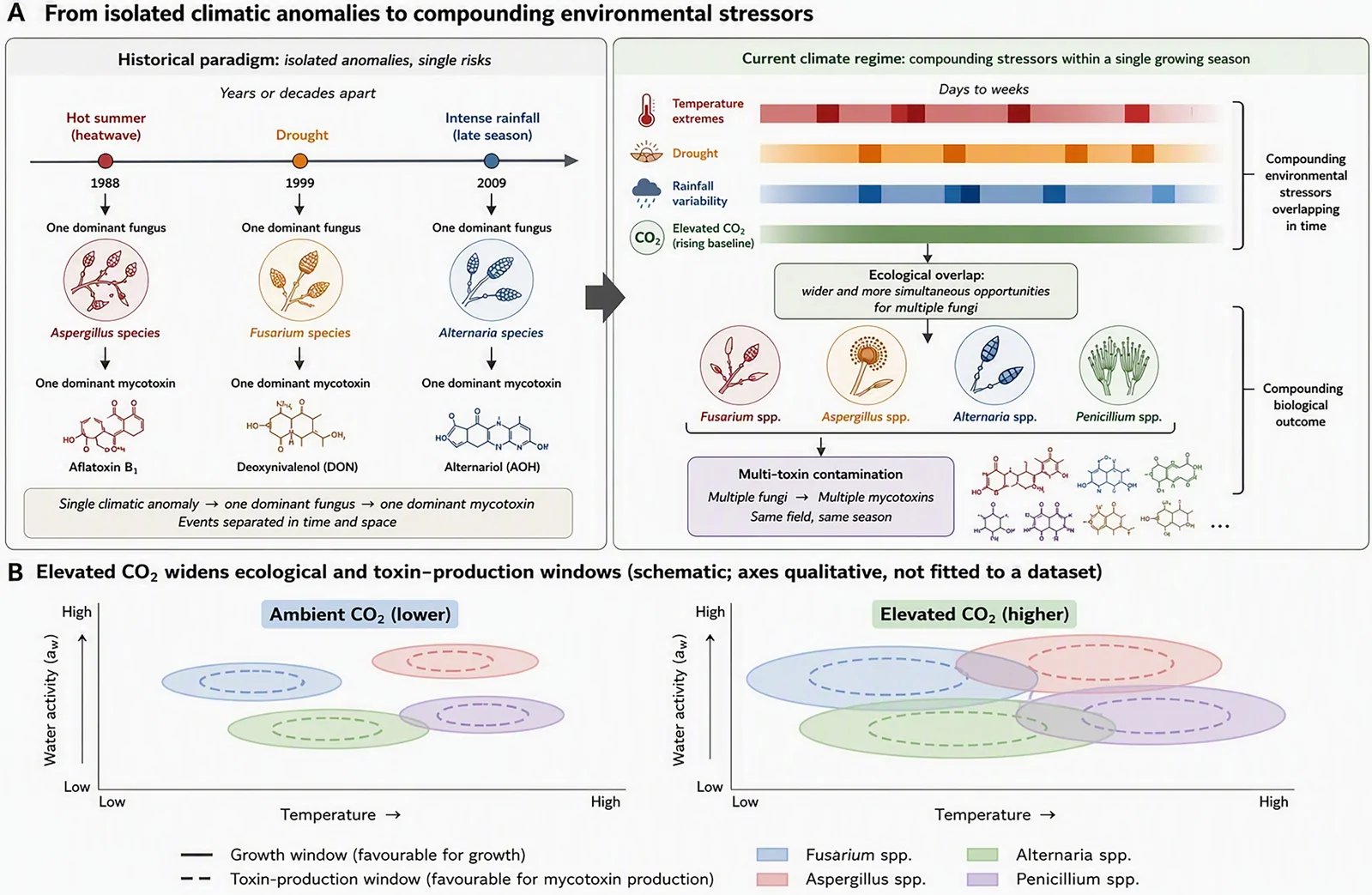
From isolated climatic anomalies to converging, multi-species contamination. **(A)** On the left, under the historical paradigm, isolated climatic anomalies, a hot summer, a drought, a late-season rainfall event, years or decades apart, were each associated with a single dominant fungal genus and a single dominant mycotoxin (*Aspergillus*/aflatoxin B1 in 1988; *Fusarium*/deoxynivalenol in 1999; *Alternaria*/alternariol in 2009). This one-fungus-one-toxin picture reflects, at least in part, the analytical and regulatory conventions of the time, when routine monitoring targeted a single regulated mycotoxin per commodity, rather than confirmed evidence that other toxins were biologically absent; the specific dates and toxin/genus pairings shown here are illustrative examples of that historical convention, not a claim about those specific years or about the underlying biology in them. On the right, under the current climate regime, temperature extremes, drought, rainfall variability and a rising CO_2_ baseline converge within days to weeks of the same growing season. This creates ecological overlap in which *Fusarium*, *Aspergillus*, *Alternaria* and *Penicillium* find simultaneously favorable conditions, consistent with multi-toxin contamination of the same field in the same season. **(B)** Elevated CO2 (~1000 ppm) is proposed to modify, and in many cases widen, both the growth window (solid outline) and the narrower, nested toxin-production window (dashed outline) of each genus relative to ambient CO_2_ (~400 ppm) across the temperature-water activity plane, and to increase the degree to which these windows overlap between genera, providing a plausible ecological basis for more frequent multi-species colonization and multi-mycotoxin contamination; the extent and direction of this shift are expected to vary among genera, species and strains, and the panel is a conceptual simplification of that variability, not a claim that every genus or species responds identically. Genus-level curves and axis ranges are schematic illustrations of the general pattern reported across species in the studies cited in Section 3, not fitted boundaries from a single quantitative dataset, and should not be read as precise numerical thresholds for any given species or study.

## 3 An ecological reframing of the growth and toxin windows

The mechanism behind this shift is ecological rather than purely meteorological. Fungal growth and mycotoxin production each have their own optimum and boundary conditions of water activity and temperature, and these two ranges are not the same (Magan and Lacey, 1988; Milani, 2013); in almost every mycotoxigenic species studied, the range permitting toxin production is narrower than the range permitting growth (Medina et al., 2017; Magan and Aldred, 2007). This is a basic distinction between the growth window of a species, the full range of conditions in which it can grow, and its toxin-production window, the narrower range in which a given trait, here toxin production, is actually expressed (terminology adapted from the fundamental/realized niche distinction of Crowther et al., 2014). Most applied mycology has, until recently, treated this as a fixed species property, measured once and used as a constant in risk models.

This treatment needs to be modified, because boundary values have traditionally been established on synthetic laboratory media, typically potato dextrose or malt extract agar, and applied as if fixed regardless of what the fungus is actually growing on. Because fungi are non-motile and feed by degrading the substrate around them, the food matrix is itself part of the environment they experience, alongside temperature, water activity and CO_2_. Growth and toxin production on a starch-rich matrix such as potato differ from those on a lipid- and protein-rich matrix such as peanut or pistachio, and boundary values measured on agar can differ considerably from those on the actual grain, nut or fruit at risk, with the toxin-permitting range again narrower on a natural substrate (Ramirez et al., 2006). Work comparing *in vitro* screening with pistachio nuts (Baazeem et al., 2021) and a grape-based matrix (Cervini et al., 2021) confirms the shift, so agar-derived boundary values start from a biased input before any climate factor is added.

Work over the past decade, including our own, has shown this is not fixed, and CO_2_ is the factor that forced this reconsideration. Adding elevated CO_2_ to the temperature-water activity interaction does not simply increase toxin output under already-favorable conditions; it widens the conditions under which toxin is produced at all, bringing the toxin-production window closer to the growth window (Medina et al., 2015; Gilbert et al., 2018; Baazeem et al., 2021) (**Figure 1B**). In *Aspergillus flavus* on maize, interacting elevated CO_2_ with drought stress and increased temperature raised aflatoxin biosynthetic gene expression (aflD, aflR) and aflatoxin B1 production, in some cases several-fold, under conditions previously inside the growth range only (Medina et al., 2015; Gilbert et al., 2018). The same pattern, growth largely unaffected while toxin production expands, recurs in pistachio-associated *A. flavus* (Baazeem et al., 2021) and grape-associated *A. carbonarius* (Cervini et al., 2021). Acclimatization over successive generations shifts this further, since strains of *Fusarium langsethiae*, *F. poae* and *F. sporotrichioides* sub-cultured for ten generations at 1000 ppm CO_2_ altered their subsequent growth and toxin-production responses relative to non-acclimatized strains, showing the window can move with repeated exposure to the same driver (Kahla et al., 2023). This evidence for a direct CO_2_ effect is strongest for *Aspergillus* systems, and confirming it across the wider fungal community remains a priority.

This is the ecological core of the argument. Climate change, acting through the interaction of temperature, water activity and CO_2_, does not only make existing conditions more frequent. It modifies, and in many cases widens, two windows that applied mycology has long treated as distinct and largely independent, the conditions under which a fungus can grow and the conditions under which it will produce toxin, though the degree and direction of this shift vary by species, strain, substrate and condition, as the pistachio and grape examples above illustrate; even where growth itself changes little, toxin production can still expand, for more species and at more points across crop development than a single-factor or single-window model would predict. This pattern has emerged from comparing growth and toxin data across species, matrices and conditions over successive studies since the mid-2000s and is the central lesson from two decades of climate change research on mycotoxigenic fungi (Magan and Medina, 2016; Medina et al., 2017).

We propose that the degree of overlap between the growth window and the toxin window offers a useful ecological metric for anticipating mycotoxin risk under climate change, one that can be estimated for a given species and matrix rather than assumed constant.

The consequence at community level matters most for contamination outcomes in the field. Where the two windows widen and move closer together for a given species, they may also increasingly overlap, at different points in time and for different species, with the equivalent windows of competing fungi. *Fusarium*, *Aspergillus*, *Penicillium* and *Alternaria* species that previously occupied separate temporal or spatial niches within a crop cycle now compete for an overlapping set of favorable conditions (Magan and Aldred, 2007), with outcomes that are strain- and species-specific rather than predictable from single-factor data alone (Cervini et al., 2021; Llobregat et al., 2025). Wider ecological windows, in other words, do not simply mean more toxin from one species; they may mean a larger, more crowded set of species with a genuine opportunity to contribute toxin to the same commodity at the same time, a plausible mechanistic basis for the multi-toxin, multi-species contamination documented in the next section.

## 4 Global evidence for the pattern

If the mechanism described above is general, not an artefact of the maize and grape systems used to establish it, its consequences should be visible well beyond those systems, in surveys never designed to test it, and the evidence reviewed below is consistent with that prediction. Patterns consistent with widening, overlapping windows have been reported across many crops and regions, in three recurring forms, geographical expansion, multi-species coexistence within a crop, and persistence along the food chain. The examples below are illustrative rather than exhaustive, and each is read here as consistent with a field-scale consequence of the same ecological mechanism, not as proof of it, and not a separate line of evidence.

### 4.1 Geographical expansion

In East Africa, household maize in Kenya carried 22 mycotoxins spanning *Aspergillus* (aflatoxins, cyclopiazonic acid) and *Fusarium* (fumonisins, zearalenone, deoxynivalenol) products, plus 31 *Aspergillus flavus* metabolites, with sharp regional differences between aflatoxin- and fumonisin-dominant areas (Kagot et al., 2022). In South America, Brazilian cocoa carried up to 15 mycotoxins from *Aspergillus*, *Penicillium* and *Fusarium* simultaneously, with emerging aflatoxin risk in cooler Chile alongside high *Fusarium* and *Alternaria* occurrence in Argentina (Foerster et al., 2024). In Europe, an 11-year survey of northern Italian maize found over 85% of samples with two or more mycotoxins from *Fusarium* and *Aspergillus* jointly (Locatelli et al., 2022), while a seven-year, 23-country survey found 52.4-74.2% co-occurrence between trichothecenes and other *Fusarium*-derived emerging mycotoxins, showing toxin diversity expanding even within a single genus (Weaver et al., 2026). Globally, a ten-year survey of 74,821 feed samples from 100 countries found 64% of samples tested for three or more mycotoxins contained at least two, most often an *Aspergillus* toxin (aflatoxin B1) paired with a *Fusarium* toxin (fumonisins) in Sub-Saharan Africa and Southeast Asia, or with an ochratoxin A-producing *Aspergillus* or *Penicillium* species in South Asia (Gruber-Dorninger et al., 2019).

### 4.2 Multi-species coexistence

The community-level widening described in Section 3 is directly visible within single crops. In maize, the shift from *Fusarium verticillioides* towards *Aspergillus flavus* dominance under hotter, drier conditions, together with the aflatoxin/cyclopiazonic acid ratio shift under interacting CO_2_ and temperature, is consistent with the broader literature on maize susceptibility under climate change (Medina et al., 2014, 2017), and with a modeling study projecting a 92% increase in aflatoxin B1 risk in European maize under a +2 °C scenario (Battilani et al., 2016). In grapes, PCR-based surveys of Spanish vineyards report *A. flavus* in 67.1%, *A. niger* in 43.5% and *A. carbonarius* in 14.1% of the same organically grown berries (Melguizo et al., 2025), three toxigenic species routinely present on the same fruit; an analogous substitution was documented earlier in northern Portugal, where *A. flavus* outcompetes *A. carbonarius* on grapes, shifting the dominant risk from ochratoxin A towards aflatoxin B1 (Serra et al., 2006a, 2006b). A similar substitution recurs on cereals, where *F. graminearum* has displaced *F. culmorum* as the head-blight pathogen of Danish wheat (Nielsen et al., 2011), and *Fusarium* head blight has intensified under warming in both southern England (West et al., 2012) and central China (Zhang et al., 2014), accompanied in China by a shift toward a more toxigenic chemotype mirroring an equivalent replacement already documented in North America (Valverde-Bogantes et al., 2019).

### 4.3 Food-chain persistence

The pattern extends beyond raw commodities and recurs across regions. In Europe, a UK survey of plant-based meat alternatives and beverages found emerging *Fusarium* mycotoxins in up to 98.9% of samples, *Alternaria* toxins in up to 90.4%, and *Aspergillus* toxins (aflatoxins up to 82.6% of meat alternatives, highest at 66.7% in legume-based products, and ochratoxin A up to 90% of soy-based beverages), spanning at least three fungal genera within the same product category (Torrijos et al., 2026). In Asia, wheat flour from Shandong, China, showed the *Fusarium* toxin deoxynivalenol with the *Alternaria* toxins tenuazonic acid and tentoxin in 78% of samples (Li et al., 2026). Co-occurrence in raw ingredients therefore carries through, and sometimes intensifies, along the food chain, not diluted out by processing.

Taken together, these three patterns are illustrative, not exhaustive, and are consistent with, though they do not on their own proof, the ecological mechanism proposed in Section 3, in which climate-driven interacting stressors would be expected to produce an increasingly common outcome that is not an elevated level of one expected toxin but the simultaneous presence of several.

Distinguishing a true increase in biological co-contamination from an increase in its detection is not straightforward, and this limitation applies throughout. Improved analytical capability, broader LC-MS/MS panels and expanded surveillance undoubtedly account for a substantial share of how much co-occurrence is now recorded (Malachova et al., 2018), and multi-mycotoxin occurrence itself is unlikely to be a wholly new phenomenon. However, the same trend appears consistently across independent surveys differing in continent, commodity and design, from an 11-year regional monitoring program in Italian maize (Locatelli et al., 2022) to a ten-year global screening of animal feed (Gruber-Dorninger et al., 2019). This consistency is compatible with a genuine biological contribution in addition to improved analytical detection, though it does not rule out that detection also contributes to the magnitude of the trend.

Climate change should therefore be viewed as a major interacting driver of these patterns rather than their sole determinant, since agronomic practices, cultivar choice, trade patterns, storage conditions, sampling strategy and analytical coverage, among others, can also shape mycotoxin outcomes and the co-occurrence recorded at every stage (Medina et al., 2017).

## 5 Why current models, monitoring and legislation fall short

**Figure 2** summarizes this proposed causal chain, from climate drivers to the resulting regulatory mismatch. Risk models, monitoring programs and regulatory limits remain built, for the most part, around single-toxin, single-species assumptions calibrated on historical, regional data (Medina et al., 2017; Casu et al., 2024), reasonable when contamination events were rare and dominated by one toxin but a poor match for several species occupying overlapping windows within the same season, especially where regulatory capacity is weakest where co-occurrence is highest (Kagot et al., 2022; Fapohunda and Adewunmi, 2019).

**Figure 2:**
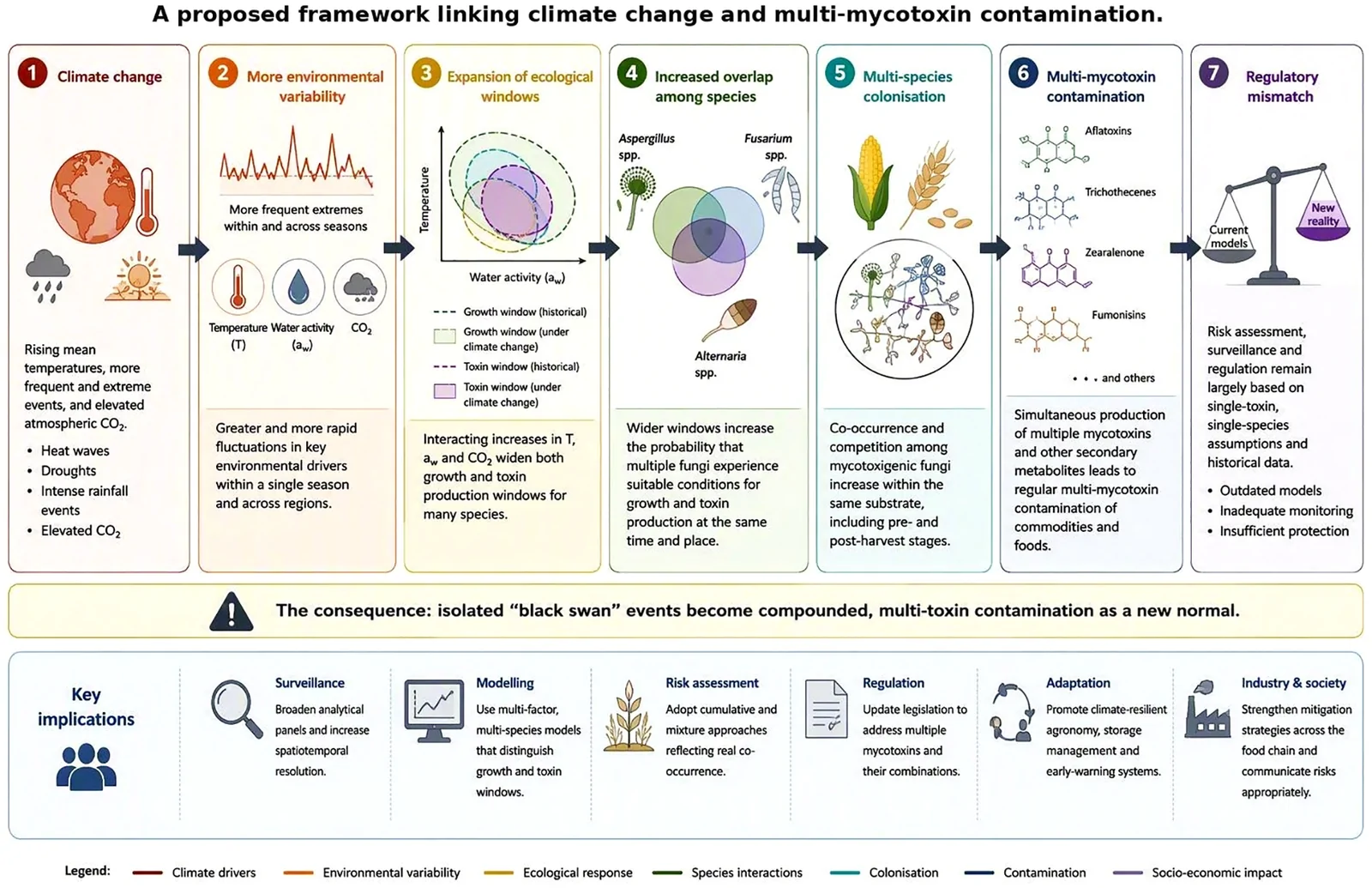
A proposed framework linking climate change and multi-mycotoxin contamination. A schematic overview of the seven-step proposed causal chain argued in this Perspective, from rising climate variability (steps 1–2) through the widening and overlap of fungal ecological windows (steps 3–4), to multi-species colonization and multi-mycotoxin contamination of commodities and foods (steps 5–6), and the resulting mismatch with risk assessment, surveillance and legislation still calibrated on single-toxin, single-species assumptions (step 7). The lower panel summarizes the practical implications for surveillance, modeling, risk assessment, regulation, adaptation and industry discussed in Sections 5 and 6. The panel is a conceptual synthesis of the argument developed in the main text.

Predictive models that treat temperature, water activity and CO_2_ as separate, additive factors will systematically underestimate risk, because it is their interaction that drives the widening of the toxin-production window described in Section 3. Legislation calibrated around single-compound thresholds does not address the toxicological risk of co-occurring mycotoxins, a gap the surveys above consistently flag as unresolved.

## 6 Toward an ecological framework for cumulative mycotoxin risk

Mycotoxin risk under climate change is better anticipated as the overlap and succession of ecological windows than as discrete trigger events.

Surveillance needs to move from single-analyte to routine multi-mycotoxin analysis and near-real-time monitoring; low-cost CO_2_ sensing has recently been proposed as an early warning signal, since CO_2_ output tracks fungal activity before toxin is detectable (Kiaitsi et al., 2025). Predictive models need to be mechanistic and multi-factor, incorporating fungal community competition, since outcomes are not predictable from single-species data (Cervini et al., 2021; Llobregat et al., 2025). Mitigation strategies validated under current conditions need re-testing under interacting future conditions, since resistance to one factor does not guarantee resistance to the combination (Llobregat et al., 2025; López-Rodríguez et al., 2025). Regulatory frameworks need to account for co-occurrence and cumulative toxicity rather than single-compound thresholds.

## 7 Discussion and concluding remarks

We propose that climate change has not simply made rare mycotoxin events more frequent but may also be changing the ecological rules determining how many toxigenic species can be active, and producing toxin, at the same time and place. This Perspective proposes a reframing in which the black swan was never disappearing but multiplying, and the mechanism behind that multiplication is ecological, not purely statistical. A warmer, more CO_2_-rich, more erratically watered world would not simply raise the odds of one rare event; the experimental evidence in Section 3 shows it widens, for the species tested, the window in which toxin production becomes possible, a mechanism we propose also operates more widely.

This has a direct consequence for how the field defines an outbreak. If ecological windows for growth and toxin production are converging for multiple species at once, the single-toxin contamination event, long treated as the default case in risk assessment, monitoring and legislation, may increasingly represent the exception rather than the rule in many production systems, as the examples reviewed here, spanning several crops, processed products and continents, already suggest. A discipline built to explain rare, single-cause events after they happen risks being poorly positioned to anticipate a regime in which layered, multi-cause, multi-toxin contamination is an increasingly common outcome rather than a surprise.

Three testable implications follow. First, if toxin-production windows continue to converge with growth windows, fungal biomass or infection level alone will increasingly under-predict toxin risk, a gap that visual inspection or generic fungal counts cannot close. Second, if species with previously separate windows now overlap, models and legislation calibrated species-by-species and toxin-by-toxin will systematically underestimate combined risk. Third, as these overlapping windows shift polewards alongside the pests and pathogens already shown to shift, regional baselines will become outdated faster than most programs are designed to detect. If this framework is correct, multi-mycotoxin contamination would be expected to rise faster than any single regulated mycotoxin across many agricultural systems, a comparison existing surveillance data could test directly.

This does not mean the underlying biology is already understood. Competition between species on a shared substrate can reshape a fungus’s biosynthetic output, more toxin, less toxin, new metabolites, or altered ratios when several compounds are produced, and how that reshaping depends on substrate and environmental conditions remains an open research question. What is not needed is new biology to justify taking the pattern seriously; the ecological and physiological evidence already available should be taken as seriously by risk models, surveillance and legislation as it already is by the researchers who generated it. The question this Perspective leaves open is not whether mycotoxin risk under climate change will keep producing surprises, given how many windows are now open at once, it almost certainly will, but whether the field keeps treating each new multi-toxin finding as another isolated black swan, or finally accepts that the flock, not the single bird, is now the more accurate description of the risk.

As anticipated in the Introduction, this widening of ecological windows also has a dimension that mycotoxin risk assessment has been slow to make explicit. The same crops that carry multi-toxin contamination into human food also, in large part, feed livestock, and the same climatic drivers, warmer temperatures, elevated CO_2_ and more erratic water availability, act simultaneously on the field, the feed store and the surrounding environment. A framework in which several toxigenic species widen their windows together, on the same substrate, at the same time, is therefore not only a food safety problem but a One Health one, since human, animal and environmental health share the same shifting hazard through shared crops, climate and exposure pathways, rather than separate, independently assessed routes (Mettenleiter et al., 2023). Treating multi-mycotoxin contamination as an isolated food-chain issue, rather than one expression of a broader climate-driven change in fungal-host-environment interactions, risks missing exposures that move between animal feed and human food, or between agricultural and adjacent natural ecosystems, before commodity-specific surveillance captures them.

If this framework is correct, surveillance programs and the regulatory bodies that rely on them, from EFSA and Codex Alimentarius to national food and feed safety agencies, should treat multi-mycotoxin contamination not as an anomaly requiring special explanation but as the baseline condition against which exceptionality is judged, calling for coordination across human, animal and environmental health sectors rather than commodity-by-commodity oversight alone.

## References

[B1] Baazeem A. Rodriguez A. Medina A. Magan N. (2021). Impacts of climate change interacting abiotic factors on growth, aflD and aflR gene expression and aflatoxin B1 production by *Aspergillus flavus* strains *in vitro* and on pistachio nuts. Toxins 13, 385. doi: 10.3390/toxins13060385 34071166 PMC8228473

[B2] Battilani P. Toscano P. van der Fels-Klerx H. J. Moretti A. Camardo Leggieri M. Brera C. . (2016). Aflatoxin B1 contamination in maize in Europe increases due to climate change. Sci. Rep. 6, 24328. doi: 10.1038/srep24328 27066906 PMC4828719

[B3] Bebber D. P. (2015). Range-expanding pests and pathogens in a warming world. Annu. Rev. Phytopathol. 53, 335–356. doi: 10.1146/annurev-phyto-080614-120207 26047565

[B4] Bebber D. P. Ramotowski M. A. T. Gurr S. J. (2013). Crop pests and pathogens move polewards in a warming world. Nat. Clim. Change 3, 985–988. doi: 10.1038/nclimate199037880705

[B5] Casu A. Camardo Leggieri M. Toscano P. Battilani P. (2024). Changing climate, shifting mycotoxins: a comprehensive review of climate change impact on mycotoxin contamination. Compr. Rev. Food Sci. Food Saf. 23, e13323. doi: 10.1111/1541-4337.13323 38477222

[B6] Cervini C. Verheecke-Vaessen C. Ferrara M. García-Cela E. Magistà D. Medina A. . (2021). Interacting climate change factors (CO_2_ and temperature cycles) effects on growth, secondary metabolite gene expression and phenotypic ochratoxin A production by *Aspergillus carbonarius* strains on a grape-based matrix. Fungal Biol. 125, 115–122. doi: 10.1016/j.funbio.2020.10.007 33518201

[B7] Chaloner T. M. Gurr S. J. Bebber D. P. (2021). Plant pathogen infection risk tracks global crop yields under climate change. Nat. Clim. Change 11, 710–715. doi: 10.1038/s41558-021-01104-837880705

[B8] Crowther T. W. Maynard D. S. Crowther T. R. Peccia J. Smith J. R. Bradford M. A. (2014). Untangling the fungal niche: the trait-based approach. Front. Microbiol. 5, 579. doi: 10.3389/fmicb.2014.00579 25400630 PMC4215788

[B9] Fapohunda S. O. Adewunmi A. A. (2019). Climate change and mycotoxins - the African experience. Croat. J. Food. Sci. Technol. 11, 283–290. doi: 10.17508/CJFST.2019.11.2.09

[B10] Foerster C. Müller-Sepúlveda A. Venturini Copetti M. Arrúa A. A. Monsalve L. Ramirez M. L. . (2024). A mini review of mycotoxin's occurrence in food in South America in the last 5 years: research gaps and challenges in a climate change era. Front. Chem. Biol. 3, 1400481. doi: 10.3389/fchbi.2024.1400481

[B11] Gilbert M. K. Medina A. Mack B. M. Lebar M. D. Rodríguez A. Bhatnagar D. . (2018). Carbon dioxide mediates the response to temperature and water activity levels in *Aspergillus flavus* during infection of maize kernels. Toxins 9, 405. doi: 10.3390/toxins9120405 29271897 PMC5793092

[B12] Gruber-Dorninger C. Jenkins T. Schatzmayr G. (2019). Global mycotoxin occurrence in feed: a ten-year survey. Toxins 11, 375. doi: 10.3390/toxins11070375 31252650 PMC6669473

[B13] Intergovernmental Panel on Climate Change (IPCC) (2022). Climate Change 2022: Impacts, Adaptation and Vulnerability. Contribution of Working Group II to the Sixth Assessment Report of the Intergovernmental Panel on Climate Change. Eds. Pörtner H.-O. Roberts D. C. Tignor M. Poloczanska E. S. Mintenbeck K. Alegría A. (Cambridge: Cambridge University Press). doi: 10.1017/9781009325844

[B14] Kagot V. De Boevre M. Landschoot S. Obiero G. Okoth S. De Saeger S. (2022). Comprehensive analysis of multiple mycotoxins and *Aspergillus flavus* metabolites in maize from Kenyan households. Int. J. Food Microbiol. 363, 109502. doi: 10.1016/j.ijfoodmicro.2021.109502 34952410

[B15] Kahla A. Verheecke-Vaessen C. Delpino-Deelias M. Gutierrez-Pozo M. Medina A. Magan N. . (2023). Acclimatisation of *Fusarium langsethiae*, *F. poae* and *F. sporotrichioides* to elevated CO_2_: impact on fungal growth and mycotoxin production on oat-based media. Int. J. Food Microbiol. 394, 110176. doi: 10.1016/j.ijfoodmicro.2023.110176 36989929

[B16] Kiaitsi E. Zanardi S. Sulyok M. Medina A. Ingram B. Suman M. . (2025). Real-time CO_2_ monitoring for early detection of grain spoilage and mycotoxin contamination. J. Sci. Food Agric. 105, 8985–8993. doi: 10.1002/jsfa.70151 40874368 PMC12595400

[B17] Li F. Wang X. Lan X. Guo X. Zhu Y. Zhang X. . (2026). Co-contamination analysis and health risk assessment of mycotoxin, metal and metalloid contaminants in wheat flour samples from Shandong, China. J. Food Meas. Charact. 20, 7248–7258. doi: 10.1007/s11694-026-04115-130311153

[B18] Llobregat B. Cervini C. González-Candelas L. Verheecke-Vaessen C. Ballester A.-R. Medina Á. (2025). Will climate change affect growth and ochratoxin A production of putative biocontrol knockout strains of *Aspergillus carbonarius*? Int. J. Food Microbiol. 439, 111250. doi: 10.1016/j.ijfoodmicro.2025.111250 40354730

[B19] Locatelli S. Scarpino V. Lanzanova C. Romano E. Reyneri A. (2022). Multi-mycotoxin long-term monitoring survey on North-Italian maize over an 11-year period, (2011-2021): the co-occurrence of regulated, masked and emerging mycotoxins and fungal metabolites. Toxins 14, 520. doi: 10.3390/toxins14080520 36006184 PMC9416020

[B20] López-Rodríguez C. Verheecke-Vaessen C. Strub C. Fontana A. Guehi T. Schorr-Galindo S. . (2025). Resilience to climate change by biocontrol yeasts against ochratoxin A production in Robusta coffee. Toxins 17, 110. doi: 10.3390/toxins17030110 40137883 PMC11945309

[B21] Magan N. Aldred D. (2007). “ Environmental fluxes and fungal interactions: maintaining a competitive edge,” in Stress in Yeasts and Filamentous Fungi. Eds. van West P. Avery S. Stratford M. ( Elsevier, Amsterdam).

[B22] Magan N. Lacey J. (1988). Ecological determinants of mould growth in stored grain. Int. J. Food Microbiol. 7, 245–256. doi: 10.1016/0168-1605(88)90043-8 3275327

[B23] Magan N. Medina A. (2016). Integrating gene expression, ecology and mycotoxin production by *Fusarium* and *Aspergillus* species in relation to interacting environmental factors. World Mycotoxin J. 9, 673–684. doi: 10.3920/WMJ2016.2076

[B24] Magan N. Medina A. Aldred D. (2011). Possible climate-change effects on mycotoxin contamination of food crops pre- and postharvest. Plant Pathol. 60, 150–163. doi: 10.1111/j.1365-3059.2010.02412.x40046247

[B25] Malachova A. Stranska M. Vaclavikova M. Elliott C. T. Black C. Meneely J. . (2018). Advanced LC-MS-based methods to study the co-occurrence and metabolization of multiple mycotoxins in cereals and cereal-based food. Anal. Bioanal. Chem. 410, 801–825. doi: 10.1007/s00216-017-0750-7 29273904 PMC5775372

[B26] Medina A. Akbar A. Baazeem A. Rodriguez A. Magan N. (2017). Climate change, food security and mycotoxins: do we know enough? Fungal Biol. Rev. 31, 143–154. doi: 10.1016/j.fbr.2017.04.00242574925

[B27] Medina A. Rodriguez A. Magan N. (2014). Effect of climate change on *Aspergillus flavus* and aflatoxin B1 production. Front. Microbiol. 5, 348. doi: 10.3389/fmicb.2014.00348 25101060 PMC4106010

[B28] Medina A. Rodriguez A. Sultan Y. Magan N. (2015). Climate change factors and *Aspergillus flavus*: effects on gene expression, growth and aflatoxin production. World Mycotoxin J. 8, 171–179. doi: 10.3920/WMJ2014.1727

[B29] Melguizo C. Tarazona A. Gil-Serna J. Mateo F. Patiño B. Mateo E. M. (2025). Toxigenic *Aspergillus* diversity and mycotoxins in organic Spanish grape berries. Toxins 17, 487. doi: 10.3390/toxins17100487 41150188 PMC12567864

[B30] Mettenleiter T. C. Markotter W. Charron D. F. Adisasmito W. B. Almuhairi S. Behravesh C. B. . (2023). The one health high-level expert panel (OHHLEP). One Health Outlook 5, 18. doi: 10.1186/s42522-023-00085-2 38062534 PMC10704771

[B31] Milani J. M. (2013). Ecological conditions affecting mycotoxin production in cereals: a review. Vet. Med. 58, 405–411. doi: 10.17221/6979-VETMED

[B32] Nielsen L. K. Jensen J. D. Nielsen G. C. Jensen J. E. Spliid N. H. Thomsen I. K. . (2011). *Fusarium* head blight of cereals in Denmark: species complex and related mycotoxins. Phytopathology 101, 960–969. doi: 10.1094/PHYTO-07-10-0188 21323468

[B33] Pritchard S. G. (2011). Soil organisms and global climate change. Plant Pathol. 60, 82–99. doi: 10.1111/j.1365-3059.2010.02405.x40046247

[B34] Ramirez M. L. Chulze S. Magan N. (2006). Temperature and water activity effects on growth and temporal deoxynivalenol production by two Argentinean strains of *Fusarium graminearum* on irradiated wheat grain. Int. J. Food Microbiol. 106, 291–296. doi: 10.1016/j.ijfoodmicro.2005.09.004 16236377

[B35] Serra R. Lourenço A. Alípio P. Venâncio A. (2006a). Influence of the region of origin on the mycobiota of grapes with emphasis on *Aspergillus* and *Penicillium* species. Mycol. Res. 110, 971–978. doi: 10.1016/j.mycres.2006.05.010 16891107

[B36] Serra R. Mendonça C. Venâncio A. (2006b). Ochratoxin A occurrence and formation in Portuguese wine grapes at various stages of maturation. Int. J. Food Microbiol. 111, S35–S39. doi: 10.1016/j.ijfoodmicro.2006.03.007 16707182

[B37] Taleb N. N. (2007). The Black Swan: The Impact of the Highly Improbable (New York: Random House).

[B38] Torrijos R. Mihalache O. A. Patriarca A. Medina A. Dall'Asta C. (2026). Mycotoxin contamination in plant-based beverages and meat alternatives: a survey of the UK market. Food Control 183, 111910. doi: 10.1016/j.foodcont.2025.11191042574925

[B39] Valverde-Bogantes E. Bianchini A. Herr J. R. Rose D. J. Wegulo S. N. Hallen-Adams H. E. (2019). Recent population changes of *Fusarium* head blight pathogens: drivers and implications. Can. J. Plant Pathol. 42, 315–329. doi: 10.1080/07060661.2019.168044237339054

[B40] Weaver A. C. Weaver D. M. de Carvalho L. V. F. M. Yiannikouris A. (2026). Influence of climatic region and feedstuff type on the co-occurrence and contamination profiles of 54 mycotoxins in European grains and forages: a seven-year survey. Toxins 18, 5. doi: 10.3390/toxins18010005 41591152 PMC12845635

[B41] West J. S. Holdgate S. Townsend J. A. Edwards S. G. Jennings P. Fitt B. D. L. (2012). Impacts of changing climate and agronomic factors on fusarium ear blight of wheat in the UK. Fungal Ecol. 5, 53–61. doi: 10.1016/j.funeco.2011.03.00342574925

[B42] Zhang X. Halder J. White R. P. Hughes D. J. Ye Z. Wang C. . (2014). Climate change increases risk of fusarium ear blight on wheat in central China. Ann. Appl. Biol. 164, 384–395. doi: 10.1111/aab.1210740046247

